# Diagnostic, Prognostic, and Therapeutic Roles of Gut Microbiota in COVID-19: A Comprehensive Systematic Review

**DOI:** 10.3389/fcimb.2022.804644

**Published:** 2022-03-04

**Authors:** Yeganeh Farsi, Azin Tahvildari, Mahta Arbabi, Fateme Vazife, Leonardo A. Sechi, Amir Hashem Shahidi Bonjar, Parnian Jamshidi, Mohammad Javad Nasiri, Mehdi Mirsaeidi

**Affiliations:** ^1^ Student Research Committee, School of Medicine, Shahid Beheshti University of Medical Sciences, Tehran, Iran; ^2^ Department of Biomedical Sciences, University of Sassari, Sassari, Italy; ^3^ Struttura Complessa (SC), Microbiologia e Virologia, Azienda Ospedaliera Universitaria, Sassari, Italy; ^4^ Clinician Scientist of Dental Materials and Restorative Dentistry, School of Dentistry, Shahid Beheshti University of Medical Sciences, Tehran, Iran; ^5^ Department of Microbiology, School of Medicine, Shahid Beheshti University of Medical Sciences, Tehran, Iran; ^6^ Division of Pulmonary and Critical Care, College of Medicine-Jacksonville, University of Florida, Jacksonville, FL, United States

**Keywords:** COVID-19, SARS-CoV-2, gastrointestinal microbiome, dysbiosis, prognosis, diagnosis, gut microbiota, therapeutic

## Abstract

**Introduction:**

The Coronavirus Disease 2019 (COVID-19) pandemic caused by Severe Acute Respiratory Coronavirus 2 (SARS-CoV-2) emerged in late December 2019. Considering the important role of gut microbiota in maturation, regulation, and induction of the immune system and subsequent inflammatory processes, it seems that evaluating the composition of gut microbiota in COVID-19 patients compared with healthy individuals may have potential value as a diagnostic and/or prognostic biomarker for the disease. Also, therapeutic interventions affecting gut microbial flora may open new horizons in the treatment of COVID-19 patients and accelerating their recovery.

**Methods:**

A systematic search was conducted for relevant studies published from December 2019 to December 2021 using Pubmed/Medline, Embase, and Scopus. Articles containing the following keywords in titles or abstracts were selected: “SARS-CoV-2” or “COVID-19” or “Coronavirus Disease 19” and “gastrointestinal microbes” or “dysbiosis” or “gut microbiota” or “gut bacteria” or “gut microbes” or “gastrointestinal microbiota”.

**Results:**

Out of 1,668 studies, 22 articles fulfilled the inclusion criteria and a total of 1,255 confirmed COVID-19 patients were examined. All included studies showed a significant association between COVID-19 and gut microbiota dysbiosis. The most alteration in bacterial composition of COVID-19 patients was depletion in genera *Ruminococcus*, *Alistipes*, *Eubacterium*, *Bifidobacterium*, *Faecalibacterium*, *Roseburia*, *Fusicathenibacter*, and *Blautia* and enrichment of *Eggerthella*, *Bacteroides*, *Actinomyces*, *Clostridium*, *Streptococcus*, *Rothia*, and *Collinsella.* Also, some gut microbiome alterations were associated with COVID-19 severity and poor prognosis including the increment of *Bacteroides*, *Parabacteroides*, *Clostridium*, *Bifidobacterium*, *Ruminococcus*, *Campylobacter*, *Rothia*, *Corynebacterium*, *Megasphaera*, *Enterococcus*, and *Aspergillus* spp. and the decrement of *Roseburia*, *Eubacterium*, *Lachnospira*, *Faecalibacterium*, and the Firmicutes/Bacteroidetes ratio.

**Conclusion:**

Our study showed a significant change of gut microbiome composition in COVID-19 patients compared with healthy individuals. This great extent of impact has proposed the gut microbiota as a potential diagnostic, prognostic, and therapeutic strategy for COVID-19. There is much evidence about this issue, and it is expected to be increased in near future.

## Introduction

A pandemic caused by Severe Acute Respiratory Coronavirus 2 (SARS-CoV-2) emerged in late December 2019 (Zhu et al., 2020). The World Health Organization (WHO) named the consequent disease as Coronavirus Disease 2019 (COVID-19) and declared it as a global emergency due to the serious public health effects (Jamshidi et al., 2021). According to the report of the WHO, until February 1, 2022, there have been about 376 million confirmed cases and about 5.6 million deaths due to COVID-19 around the world.

The angiotensin-converting enzyme 2 (ACE2) receptor is a known SARS-CoV-2 receptor for entering host cells (Li et al., 2003; Zhou et al., 2020). This receptor is detected in various cells of the body such as the respiratory, digestive, renal, and skin epithelium, suggesting that each of these organs could be a potential target for the virus (Jamshidi et al., 2021; Xue et al., 2021). Moreover, virus RNA and viral particles have been identified in the fecal sample of COVID-19 patients, which may indicate the possibility of virus replication and activity in the human intestine (Gu et al., 2020a; Lamers et al., 2020; Xu et al., 2020).

Gut microbiota plays a well-known role in regulating immune system responses (Donaldson et al., 2016;; Schirmer et al., 2016). Recent studies indicate the role of gut dysbiosis in the pathogenesis of various diseases such as inflammatory bowel disease, type 1 and type 2 diabetes, and celiac disease, as well as chronic respiratory diseases such as asthma, COPD, and cystic fibrosis (Jamshidi et al., 2019; Enaud et al., 2020).

Bacteria in the human intestinal flora appear to affect the respiratory system and lungs (especially the lung microbiota) by producing metabolites, endotoxins, cytokines, and intestinal hormones reaching the bloodstream, which is called the gut–lung axis (Budden et al., 2017; Dang and Marsland, 2019; Zhang et al., 2020).

On the other hand, there is evidence of the role of gut dysbiosis in the severity and prognosis of bacterial (e.g., *Streptococcus pneumonia*, *Klebsiella pneumonia*, *Pseudomonas aeruginosa*, *Mycobacterium tuberculosis*) and viral (e.g., H1N1 influenza) respiratory infectious diseases in animal models (Ichinohe et al., 2011; Fagundes et al., 2012; Fox et al., 2012; Brown et al., 2017). The use of broad-spectrum antibiotics that target the gut microbiota has led to a poor prognosis in mouse models with infectious lung diseases (Enaud et al., 2020).

Considering the important role of gut microbiota in maturation, regulation, and induction of the immune system and subsequent inflammatory processes, it seems that evaluating the composition of gut microbiota in COVID-19 patients compared with healthy individuals may have potential value as a diagnostic and/or prognostic biomarker of the disease. Also, therapeutic interventions affecting gut microbial flora may open new horizons in the treatment of COVID-19 patients and accelerating their recovery.

## Methods

This review conforms to the “Preferred Reporting Items for Systematic Reviews and Meta-Analyses” (PRISMA) statement (Moher et al., 2009).

### Search Strategy and Selection Criteria

To investigate the diagnostic, prognostic, and therapeutic role of the gut microbiota composition in COVID-19, a systematic search was conducted for relevant studies published from December 2019 to December 2021 using Pubmed/Medline, Embase, and Scopus.

Articles containing the following keywords in titles or abstracts were selected: “SARS-CoV-2” or “COVID-19” or “Coronavirus Disease 19” and “gastrointestinal microbes” or “dysbiosis” or “gut microbiota” or “gut bacteria” or “gut microbes” or “gastrointestinal microbiota”. Only studies included if they contained data about the gut microbiota composition in COVID-19 patients. There were no language restrictions. Review articles, duplicate publications, letters, commentary, animal studies, and articles with no relevant data were excluded from the analysis. Two authors (MA and FV) independently screened the articles by title and abstract. Full-text screening was conducted by two other authors independently (AT and YF). In each step, contrarieties were discussed with a third reviewer (PJ).

### Data Extraction

A data extraction form designed by two authors (PJ and MJN) and, finally, selected data were extracted from the full text of eligible publications by PJ, YF, AT, MA, and FV. The following data were extracted for further analysis: first author’s name, year of publication, country where the study was executed, type of study, study population, mean age, gender, COVID-19 severity of the cases, comorbidity(ies), microbiota analysis technique, intestinal microbiota alterations, biochemical and immunological alterations, and studied value of gut microbiota alterations in COVID-19. The data were jointly reconciled, and disagreements were discussed and resolved by review authors (PJ, MJN).

### Quality Assessment

The critical appraisal checklist for case reports provided by the Joanna Briggs Institute (JBI) was used to perform a quality assessment of the studies (Institute, 2021).

## Results

**Figure 1:**
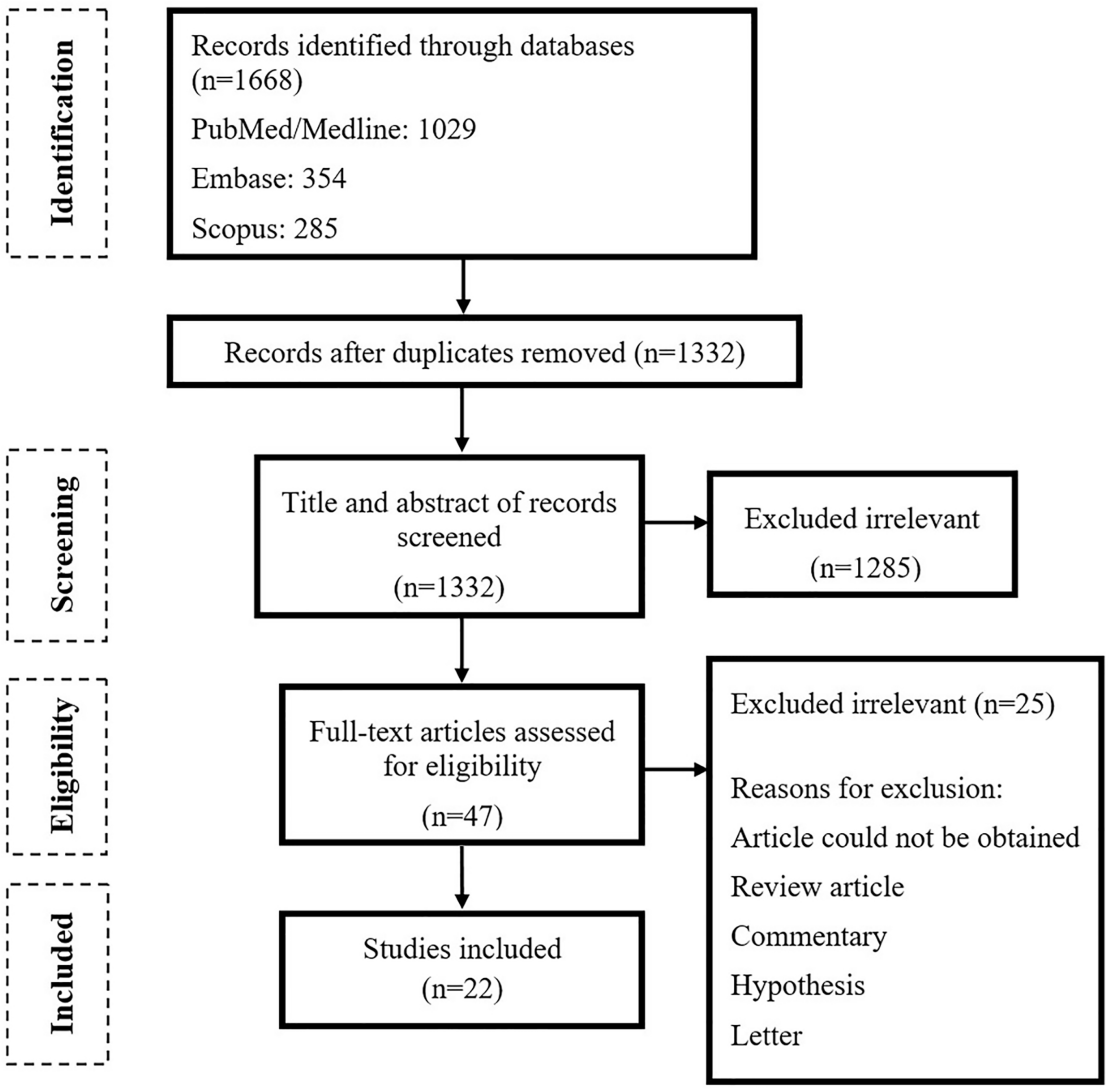
PRISMA flowchart of study selection for inclusion in the systematic review.

As shown in **Figure 1**, the primary search resulted in 1,668 relevant articles, of which 47 articles were selected after title and abstract screening. Following the full-text screening, 22 articles fulfilled the inclusion criteria. Most of the studies were case–control (n = 9) followed by cohort (n = 9), clinical trial (n = 2), and cross-sectional (n = 2) studies. Fifteen of the studies were executed in China, 2 in Italy and 1 in UK, Portugal, India, Egypt, and Korea (**Table 1**).

**Table 1:** Characteristics of the included studies.

Authors	Year	Country	Type of study	Study population	Gut microbiota analysis technique
Gu et al. (2020b)	2020	China	Cross-sectional	30 COVID-19, 24 H1N1, 30 HC*^a^*	16S rRNA sequencing
d’Ettorre et al. (2020)	2020	Italy	Clinical trial	70 COVID-19 (case: 28, control: 42)*^b^*	NM
Tang et al. (2020)	2020	China	Cohort	57 COVID-19 (20 non-severe, 19 severe, 18 critical)	q- PCR
Zuo et al. (2020b)	2020	China	Case–control	30 cases (COVID-19), 39 control (30 HC*^a^* and 9 CAP*^c^*)	Shotgun metagenomic sequencing
Zuo et al. (2020b)	2020	China	Case–control	15 cases (COVID-19), 21 control (15 HC*^a^* and 6 CAP*^c^*)	Shotgun metagenomic sequencing and RT-PCR
Zuo et al. (2021a)	2020	China	Cohort	15 COVID-19	Shotgun metagenomic sequencing
Cao et al. (2021)	2021	China	Cohort	13 COVID-19, 5 HC*^a^*	cDNA sequencing, bacteriome sequencing, metagenomic sequencing
Liu et al. (2021)	2021	China	Clinical trial	11 COVID-19	16s rRNA sequencing
Lv et al. (2021b)	2021	China	Cohort	67 COVID-19, 35 H1N1, 48 HC*^a^*	q-PCR on DNA extract of fecal samples
Lv et al. (2021a)	2021	China	Cohort	56 COVID-19, 47 HC*^a^*	NM
Yeoh et al. (2021)	2021	China	Cohort	100 COVID-19, 78 non-COVID-19	Shotgun sequencing of DNA extracted from stools
Prasad et al. (2021)	2021	UK	Cohort	30 COVID-19, 16 HC*^a^*	16S rRNA sequencing, metatranscriptomic analysis (plasma samples)
He et al. (2021)	2021	China	Case–control	13 cases (COVID-19), 21 control (HC*^a^*)	Metaproteomics
Zhou et al. (2021)	2021	China	Case–control	15 cases (recovered COVID-19 patients), 14 control (HC*^a^*)	16S rRNA sequencing
Moreira-Rosário et al. (2021)	2021	Portugal	Cross-sectional	115 COVID-19 (19 mild, 37 moderate, 58 severe)	16S rRNA sequencing
Wu et al. (2021)	2021	China	Case–control	53 cases (COVID-19), 76 control (HC*^a^*)	16S rRNA sequencing
Gaibani et al. (2021)	2021	Italy	Case–control	STUDY1: 69 COVID-19, 69 HC*^a^* STUDY2: 69 COVID-19, 16 non-COVID-19 ICU admitted control	16S rRNA sequencing
Kim et al. (2021)	2021	Korea	Case–control	12 cases (COVID-19), 36 control (HC*^a^*)	16S rRNA sequencing
Zuo et al. (2021b)	2021	China	Case–control	98 cases (COVID-19), 78 control (HC*^a^*)	Shotgun metagenomic sequencing
Khan et al. (2021)	2021	India	Case–control	30 cases (COVID-19), 10 control(HC*^a^*)	16S rRNA sequencing
Hegazy et al. (2021)	2021	Egypt	Cohort	200 COVID-19 (122 mild, 78 moderate)	NM
Wang et al. (2021b)	2021	China	Cohort	156 COVID-19 (98 mild and moderate, 58 severe and critical)*^d^*	NM

^a^Healthy control subjects.

^b^42 patients received hydroxychloroquine, antibiotics, and tocilizumab, alone or in combination, and 28 patients received the same therapy added with oral bacteriotherapy, using a multistrain formulation.

^c^Community-acquired pneumonia.

^d^The efficacy of probiotic treatment has been studied only in 16 severe and critical COVID-19 patients (treatment group = 10, control group = 6).

A total of 1,255 confirmed COVID-19 patients were examined in 22 included articles (**Table 1**). Among the COVID-19 patients whose comorbidity was mentioned by the authors, the most common ones were hypertension (32.8%) and diabetes mellitus (17.8%). Other reported comorbidities were chronic respiratory disease (6.9%), cardiovascular disease (3%), immunosuppression (2.9%), dyslipidemia (2.3%), thrombotic events (2%), and renal impairment (1.6%). See **Table 2**.

**Table 2:** Characteristics of the study population.

Authors	Age (Mean)	Gender	COVID-19 Severity	Comorbidity
Gu et al. (2020b)	52.3 years	49M, 35F	15 non-severe, 15 severe	HTN (H1N1: 5/24, COVID-19: 9/30)
d’Ettorre et al. (2020)	59.75 years	41M, 29F	70 severe: stage III *^a^*	None
Tang et al. (2020)	64.17 years	29M, 28F	20 non-severe, 19 severe, 18 critical*^b^*	HTN: 7 in non-severe, 8 in severe, 12 in critical DM: 3 in non-severe, 0 in severe, 6 in critical
Zuo et al. (2020b)	43 years	36M, 33F	NM	11 in COVID-19, 9 in CAP, 0 in healthy…not specified
Zuo et al. (2020a)	51.25 years	20M, 16F	NM	6 in COVID-19, 6 in CAP, 0 in healthy…not specified
Zuo et al. (2021a)	53 years	7M, 8F	11 moderate to severe, 2 critical (ICU)	HTN: 4, DM: 2, hyperlipidemia: 4, obesity: 1, chronic hepatitis B: 1, renal impairment: 1, duodenal ulcer: 1, left subclavian artery occlusion: 1
Cao et al. (2021)	48 years	6M, 7F	3 severe, 7 moderate, 3 mild	HTN:4, hyperthyroidism:1, gallstone:1, arthritis:1
Liu et al. (2021)	49.8 years	6M, 5F	10 non-severe, 1 severe	None
Lv et al. (2021b)	52 years	92M, 58F	36 severe	HTN: 16, diabetes: 10, CVD: 4, liver diseases: 2
Lv et al. (2021a)	54 years	57M, 46F	26 mild, 30 severe	HTN: 18, DM: 7
Yeoh et al. (2021)	41 years	86M, 92F	47 mild, 45 moderate, 5 severe, 3 critical	HTN: 11, hyperlipidemia: 4, DM: 2, CVD: 2, allergic disorders: 7, HIV: 3, asthma: 2
Prasad et al. (2021)	52 years	23M, 23F	11 mild, 17 moderate, 2 severe	DM: 10, thrombotic events: 15
He et al. (2021)	37.9 years	22M, 12F	7 mild, 5 moderate, 1 severe	DM: 1, sinusitis: 1,rhinitis:1
Zhou et al. (2021)	33.1 years	8M, 21F	NM	HTN: 2
Moreira-Rosário et al. (2021)	68 years	73M, 42F	19 mild, 37 moderate, 58 severe	DM: 45, HTN: 67, chronic respiratory disease: 21, immunosuppression: 11, hematological-oncological disease: 9
Wu et al. (2021)	48.5 years	82M, 46F*^c^*	30 non-severe, 20 Severe	NM
Gaibani et al. (2021)	73 years	38M, 31F*^d^*	NM	HTN: 44, DM: 12, CVD: 5, immunosuppression: 7, CKD:11
Kim et al. (2021)	26 years	8M, 4F*^d^*	12 asymptomatic or mild	NM
Zuo et al. (2021b)	33 years	85M, 91F	3 asymptomatic, 53 mild, 34 moderate, 5 severe, 3 critical	55… not specified
Khan et al. (2021)	51.8 years	NM	NM	NM
Hegazy et al. (2021)	41 years	94M,106 F	122 mild, 78 moderate	DM: 30, HTN: 34, chronic lung disease: 15, chronic liver disease: 2, CVD:9
Wang et al. (2021b)	48.5 years	95M, 61F	98 mild and moderate, 58 severe and critical	DM: 18, HTN: 31, CVD:15, COPD:12

NM, not mentioned; HTN, hypertension; DM, diabetes mellitus; CVD, cardiovascular disease; CKD, chronic kidney disease, COPD, chronic obstructive pulmonary disease.

^a^According to the syndromic classification proposed by the Italian Society of Anesthesia and Resuscitation (SIAARTI).

^b^Respiratory failure requiring mechanical ventilation, shock, or other organ failure requiring ICU care.

^c^There was one missing data among COVID-19 group.

^d^Only the data of the COVID-19 group was available.

Ten studies assessed gut microbiota composition alteration by fecal samples; one study used plasma samples, and it was not mentioned by the rest. The most commonly used techniques in these studies for detection and assessment of gut microbiota were 16s rRNA sequencing and shotgun metagenomic sequencing analysis (**Table 1**).

### Gut Microbiome Dysbiosis of COVID-19 Patients

All included studies showed a significant association between COVID-19 and gut microbiota dysbiosis (**Table 3**). The most alteration in the bacterial composition of COVID-19 patients was depletion in genera *Ruminococcus*, *Alistipes*, *Eubacterium*, *Bifidobacterium*, *Faecalibacterium*, *Roseburia*, *Fusicathenibacter*, and *Blautia* and enrichment of *Eggerthella*, *Bacteroides*, *Actinomyces*, *Clostridium*, *Streptococcus*, *Rotia*, and *Collinsella.* Details are shown in **Table 4**.

**Table 3:** Association between gut microbiota and COVID-19.

Authors	Type of study	Studied value	Association between gut microbiota and COVID-19
Gu et al. (2020b)	Cross-sectional	Diagnostic	Yes
d’Ettorre et al. (2020)	Clinical trial	Therapeutic	Yes
Tang et al. (2020)	Cohort	Prognostic and diagnostic	Yes
Zuo et al. (2020b)	Case–control	None	Yes
Zuo et al. (2020a)	Case–control	Prognostic and therapeutic	Yes
Zuo et al. (2021a)	Cohort	Prognostic	Yes
Cao et al. (2021)	Cohort	Diagnostic and prognostic	Yes
Liu et al. (2021)	Clinical trial	Therapeutic (postinfection recovery)	Yes
Lv et al. (2021b)	Cohort	Diagnostic	Yes
Lv et al. (2021a)	Cohort	Diagnostic and prognostic	Yes
Yeoh et al. (2021)	Cohort	Prognostic	Yes
Prasad et al. (2021)	Cohort	Diagnostic and prognostic	Yes
He et al. (2021)	Case–control	None	Yes
Zhou et al. (2021)	Case–control	None	Yes
Moreira-Rosário et al. (2021)	Cross-sectional	Prognostic	Yes
Wu et al. (2021)	Case–control	Diagnostic and prognostic	Yes
Gaibani et al. (2021)	Case–control	Prognostic	Yes
Kim et al. (2021)	Case–control	Diagnostic (postinfection recovery)	Yes
Zuo et al. (2021b)	Case–control	Diagnostic and prognostic	Yes
Khan et al. (2021)	Case–control	Prognostic	Yes
Hegazy et al. (2021)	Cohort	Prognostic	Yes
Wang et al. (2021b)	Cohort	Therapeutic	Yes

**Table 4:** Gut microbiota alterations.

Authors	Intestinal microbial alternations
Gu et al. (2020b)	**COVID-19 and H1N1 vs. HC*^a^*:** Microbial diversity↓, *Streptococcus spp.*↑, *Escherichia-Shigella spp.*↑ **H1N1 vs. COVID-19 and HC:** phylum (Actinobacteria↓, Firmicutes↓), class (Actinobacteria↓, Erysipelotrichia↓, Clostridia↓), family (Lachnospiraceae↓, Ruminococcaceae↓), *Blautia spp.*↓, *Agathobacter spp.*↓, *Anaerostipes spp.*↓, *Fusicatenibacter spp.*↓, *Eubacterium hallii* group↓, unclassified Lachnospiraceae↓, *Dorea spp.*↓, *Faecalibacterium spp.*↓, *Ruminococcus-2 spp.*↓ **COVID-19 vs. HC:** Ruminococcaceae UCG-013↓, *Roseburia spp.*↓, Lachnospiraceae family↓(*Fusicatenibacter spp.*↓, *Anaerostipes spp.*↓, *Agathobacter spp.*↓, unclassified Lachnospiraceae↓, *Eubacteriumhallii* group↓), *Streptococcus spp.*↑↑ **COVID-19 vs. H1N1:** *Streptococcus spp.* ↑↑, *Prevotella spp.*↓, *Ezakiella spp.*↓, *Murdochiella spp.*↓, *Porphyromonas spp.*↓ **COVID-19 dominated by:** *Streptococcus spp.*, *Rothia spp.*, *Veillonella spp.*, *Erysipelato clostridium spp.*, *Actinomyces spp.* **HC dominated by:** *Bifidobacterium spp.*, *Romboutsia spp.*, *Faecalibacterium spp.*, *Fusicatenibacter spp.*, *Eubacterium hallii* group, *Blautia spp.*, *Collinsella spp.* **H1N1 dominated by:** *Enterococcus spp.*, *Prevotella spp.*, *Finegoldia spp.*, *Peptoniphilus spp.*Richness, diversity, and structure of the gut microbiota were not significantly different between general and severe COVID-19 patients.
d’Ettorre et al. (2020)	The formulation administered in this study contained: *Streptococcus thermophilus* DSM 32345, *Lactobacillus acidophilus* DSM 32241, *Lactobacillus helveticus* DSM 32242, *Lactobacillus paracasei* DSM 32243, *Lactiplantibacillus plantarum* DSM 32244, *Lactobacillus brevis* DSM 27961, *Bifidobacterium lactis* DSM 32246, *Bifidobacterium lactis* DSM 32247.
Tang et al. (2020)	*Lactobacillus spp.*↓, *Bifidobacterium spp.*↓, *Faecalibacterium prausnitzii*↓, *Clostridium butyricum*↓, *Clostridium leptum*↓, *Eubacterium rectale*↓, Enterobacteriaceae ↓, *Bacteroides spp.*↓*^b^Enterococcus spp.*↑ (It was increased with disease severity)
Zuo et al. (2020b)	*Candida albicans*↑, *Aspergillus flavus*↑, *Aspergillus niger*↑
Zuo et al. (2020a)	**Antibiotic negative COVID-19 group:** *Clostridium hathewayi*↑, *Actinomyces viscosus*↑, *Bacteroides nordii*↑ **Antibiotic positive COVID-19 group:** *Faecalibacterium prausnitzii*↓, *Lachnospiraceae* bacterium↓, *Eubacterium rectale*↓, *Ruminococcus obeum*↓, *Dorea formicigenerans*↓
Zuo et al. (2021a)	**Enriched in fecal samples in high infectivity:** *Collinsella aerrofaciens*↑, *Morganella morganii*↑, *Streptococcus infantis*↑ **Enriched in fecal samples in low to non infectivity:** *Parabacteroides merdae*↑, *Bacteroides stercoris*↑, *Alistipes onderdonkii* ↑, *Lachnospiraceae* bacterium↑
Cao et al. (2021)	**COVID-19 vs. HC:Bacteria:** *Ruminococcus gnavus*↑*, Eggerthella spp.*↑*, Coprobacillus spp.*↑, *Lachnospiraceae* bacterium 2_1_58FAA↑, *Clostridium ramosum*↑*, Eggerthella lenta*↑, Lachnospiraceae bacterium 1_4_56FAA↑, *Alistipes_sp_AP11*↓, *Roseburia intestinalis*↓, Burkholderiales bacterium 1_1_47↓, *Eubacterium_hallii*↓, *Parasutterella_excrementihominis*↓, *Alistipes indistinctus*↓, *Coprobacter fastidiosus*↓, *Eubacterium eligens*↓, Bacterioidales bacterium ph8↓, *Bacterioides salyersiae*↓, *Odoribacter splanchnicus*↓, *Alistipes shahii*↓, *Ruminococcus bromii*↓, *Bacteroides massiliensis*↓ **Virome:** Inviridae↑, Microviridae↑, virgaviridae↑ **Antibiotic positive vs. antibiotic negative COVID-19 patients:Bacteria:** *Subdoligranulum*↓, *Roseburia inulinivorans*↓, *Roseburia hominis*↓, *Parasutterella excrementihominis*↓, *Lachnospiraceae* bacterium 2_1_46FAA↓, *Faecalibacterium prausnitzii*↓,*Dorea formicigenerans*↓, *Coprococcus catus*↓, *Collinsella aerofaciens*↓, *Bacteroides vulgatus*↓,*Veillonella parvula*↑, *Coprobacillus spp.*↑, *Clostridium ramosum*↑ **Virome:** No virus was identified as a differential species. **Effect of disease severity on gut microbiome:** **Virome:** Severe cases: Fourteen Microviridaephages, one Inoviridae phage, one Podoviridae phage and one unclassified virus↑ Mild cases: No viral community increased **Bacteria:** Severe cases: *Corynebacterium durum*↑, *Rothia mucilaginosa*↑, *Enterococcus faecium*↑, *Campylobacter gracilis*↑, *Corynebacterium spp.*↑, *Enterococcus spp.*↑, *Rothia spp.*↑, *Megasphaera spp.*↑, *Campylobacter spp.*↑, *Eubacterium spp.*↓ Mild cases: *Eubacterium rectale*↑
Liu et al. (2021)	**After intervention(FMT):** Proteobacteria ↓, Actinobacteria ↑, *Bifidobacterium spp.*↑, *Faecalibacterium spp.*↑, *Collinsella spp.*↑
Lv et al. (2021b)	**COVID-19 vs. others:** *Candida glabrata*↓, *Candida parapsilosis*↓, Five unclassified species separately belonging to Helotiales↓, Pleosporales↓, Sordariales↓, *Microscypha spp.*↓,*Emericellopsis spp.*↓, *Cystobasidium spp.*↑, An unclassified species of Exidiaceae↓, *Trebouxiadecolorans*↓, An unclassified species belonging to the kingdom Chromista↓
Lv et al. (2021a)	**COVID-19 vs. others:** Ruminococcaceae↓, *Eubacteriumhallii* group↓, Family XIII AD3011 group↓, *Anaerostipes spp.*↓, *Fusicatenibacter spp.*↓, *Roseburia spp.*↓, *Faecalibacterium spp.*↓, *Ruminococcus spp.* 5139BFAA↓, *Aspergillus rugulosus*↓, *Aspergillus tritici*↓,*Penicillium spp.*↓, *Penicillium citrinum* ↓, *Actinomyces spp.*↑, *Sphingomonas spp.*↑, *Rothia spp.*↑, *Actinomyces odontolyticus*↑, *Streptococcus parasanguinis*↑, *Aspergillus penicillioide*↓
Yeoh et al. (2021)	*Ruminococcus gnavus*↑, *Bacteroides dorei*↑, *Ruminococcus torques*↑, *Bacteroides vulgates*↑, *Bacteroides ovatus*↑, *Bacteroides caccae*↑,*Akkermansia muciniphila*↑, *Bifidobacterium adolescentis*↑, *Eubacterium rectal*↑, *Ruminococcus bromii*↑, *Subdoligranulum unclassified*↑, *Bifidobacterium pseudocatenulatum*↑, *Faecalibacterium prausnitzii*↑, *Collinsella aerofaciens*↑, *Ruminococcus obeum*↑, *Dorea longicatena*↑, *Coprococcus comes*↑, *Dorea formicigenerans*↑
Prasad et al. (2021)	**Plasma samples:** Proteobacteria↑, Firmicutes ↑, Actinobacteria ↑, *Acinetobacter spp.*↑, *Nitrospirillum spp.*↑, *Cupriavidus spp.*↑, *Pseudomonas spp.*↑, *Aquabacterium spp.*↑, *Burkholderia spp.*↑, *Caballeronia spp.*↑, *Paraburkholderia spp.*↑, *Bravibacterium spp.*↑, *Sphingomonas spp.*↑, *Staphylococcus spp.*↓, *Lactobacillus spp.*↓
He et al. (2021)	*Ruminococcus gnavus*↓, Lachnospiraceae↓, *Tyzzerella spp.*↓, *Blautia spp.*↓, *Eubacterium spp.*↓, Peptostreptococcaceae↓,*Butyrivibrio spp.*↓, *Ruminococcus spp.*↓, *Lachnoclostridium spp.*↓, *Bacteroides uniformis*↑, *Bacteoides graminisolvens*↑, *Bacteroides coprophilus*↑
Zhou et al. (2021)	Phylum (Actinobacteria↑), Family (Lachnospiraceae↓, Desulfovibrionaceae↓), *Faecalibacterium spp.*↓, *Roseburia spp.*↓, *Fusicatenibacter spp.*↓, *Ruminococcus spp.*↓, *Clostridium* XVIII↓, *Dorea spp.*↓, *Butyricicoccus spp.*↓, *Romboutsia spp.*↓, *Intestinimonas spp.*↓, *Bilophila spp.*↓, *Escherichia spp.*↑, *Flavonifractor spp.*↑, *Intestinibacter spp.*↑, *Intestinibacter bartlettii*↑, *Clostridium aldenense*↑, *Clostridium bolteae*↑, *Flavonifractor plautii*↑, *Clostridium ramosum*↑, *Faecalibacterium prausnitzii*↓, *Roseburia inulinivorans*↓, *Fusicatenibacter saccharivorans*↓, *Ruminococcus bromii*↓, *Blautia faecis*↓, *Butyricicoccus pullicaecorum*↓, *Intestinimonas butyriciproducens*↓
Moreira-Rosário et al. (2021)	**Severe cases:** Proteobacteria↑, Firmicutes/Bacteroidetes ratio↓, *Roseburia spp.*↓, *Lachnospira spp.*↓
Wu et al. (2021)	*Blautia spp.*↓, *Coprococcus spp.*↓, *Collinsella spp.*↓, *Streptococcus spp.*↑, *Weissella spp.*↑, *Enterococcus spp.*↑, *Rothia spp.*↑, *Lactobacillus spp.*↑, *Actinomyces spp.*↑, *Granulicatella spp.*↑, *Bacteroides caccae*↓, *Bacteroides coprophilus*↓, *Blautia obeum*↓, *Clostridium colinum*↓, *Clostridium citroniae*↑, *Bifidobacterium longum*↑, *Rothia mucilaginosa*↑ **Associations between gut microbiota disturbance and SARS-CoV-2 viral loads:** *Prevotella copri* and *Eubacterium dolichum* were positively correlated and *Streptococcus anginosus*, *Dialister spp.*, *Alistipes spp.*, *Ruminococcus spp.*, *Clostridium citroniae*, *Bifidobacterium spp.*, *Haemophilus spp.*, and *Haemophilus parainfluenzae* were negatively correlated with the viral load of SARS-CoV-2.
Gaibani et al. (2021)	**Study1*^c^*:** Enterococcaceae↑, Coriobacteriaceae↑, Lactobacillaceae↑, Veillonellaceae↑, Porphyromonadaceae↑, Staphylococcaceae↑, Bacteroidaceae↓, Lachnospiraceae↓, Ruminococcaceae↓,Prevotellaceae↓, Clostridiaceae↓ **Study2*^c^*:** *Enterococcus spp.*↑, *Klebsiella spp.*↓, *Ruminococcus spp.*↑
Kim et al. (2021)	*Escherichia spp.*↑, *Citrobacter spp.*↑, *Collinsella spp.*↑, *Bifidobacterium spp.*↑, *Bacteroides spp.*↓, *Butyricimonas spp.*↓, *Odoribacter spp.*↓
Zuo et al. (2021b)	Pepper Mild Mottle Virus (PMMoV)↓, Eukaryotic viruses particularly environment-derived eukaryotic viruses with unknown host↑, Streptococcus phage↑, Escherichia phage↑, Homavirus↑, Lactococcus phage↑, Ralstonia phage↑, Solumvirus↑, Microcystis phage↑ **Severe cases:** plant-derived RNA virus↓, Pepper Chlorotic Spot Virus (PCSV)↓, Myxococcus phage↓, Rheinheimera phage↓, Microcystis virus↓, Bacteroides phage↓, Murmansk poxvirus↓, Saudi moumouvirus↓, Sphaerotilus phage↓, Tomelloso virus↓, Ruegeria phage↓
Khan et al. (2021)	Firmicutes↓, Bacteroidetes↑, Proteobacteria↑, Actinobacteria↑**Severe cases:** *Bacteroides plebeius*↓, *Faecalibacterium prausnitzii*↓, *Roseburia faecis*↓, *Bifidobacterium spp.*↑, *Bacteroides caccae*↑, *Bacteroides ovatus*↑, *Bacteroides fragilis*↑, *Ruminococcus gnavus*↑, *Clostridium bolteae*↑, *Clostridium citroniae*↑, *Clostridium hathewayi*↑, *Parabacteroides distasonis*↑
Hegazy et al. (2021)	The formulation used in this study was a yogurt containing *Bifidobacterium spp.* and *Lactobacillus spp.*
Wang et al. (2021b)	Probiotic administration protocol: Bifidobacterium lactobacillus triplex live tablet; each tablet contained no less than 0.5×107 CFU of live *Bifidobacterium longum*; live *Lactobacillus bulgaricus* and *Streptococcus thermophilus* that was not lower than 0.5×106 CFU, 4 pieces at a time, 3 times a day.

^a^Healthy control.

^b^Bacteroides spp. was decreased in all groups, but the decrease was within the lower normal limits. There was no significant difference between the groups.

^c^Study1: comparison between COVID-19 patients and healthy controls, Study2: comparison between COVID-19 patients and non-COVID-19 ICU admitted control.

Three articles surveyed the gut mycobiota alterations, and different results have been reported for different species of the same genus. About the *Candida* spp., an increase in *Candida albicans* and a decrease in *Candida glabrata* and *Candida parapsilosis* were mentioned. In regard to *Aspergillus* spp., enrichment of *Aspergillus flavus* and *Aspergillus niger* and a depletion of *Aspergillus rugulosus*, *Aspergillus tritici*, and *Aspergillus penicillioides* were reported. Also, one study indicated a reduction in seven unclassified species belonging to order Helotiales, Pleosporales, and Sordariales, family Exidiaceae, and genera *Microscypha* and *Emericellopsis* in COVID-19 patients (**Table 4**).

According to all gut microbiota changes that were mentioned in the reviewed articles, a decrease in phyla Firmicutes and Bacteroidetes and an increase in phylum Actinobacteria among COVID-19 patients were inferred.

### Association Between Gut Microbiota Composition and COVID-19 Severity

A few studies indicated the role of gut microbiome in COVID-19 severity (**Table 4**). In severe COVID-19 cases, *Bacteroides* spp., *Parabacteroides* spp., *Clostridium* spp., *Bifidobacterium* spp., *Ruminococcus* spp., *Campylobacter* spp., *Rothia* spp., *Corynebacterium* spp., *Megasphaera* spp., *Enterococcus* spp., and *Aspergillus* spp. were increased and *Roseburia* spp., *Eubacterium* spp., *Lachnospira* spp., *Faecalibacterium* spp., and Firmicutes/Bacteroidetes ratio were decreased significantly. In subjects with mild disease the observed significant change was in the enrichment of *Eubacterium* spp.

The alteration of the gut virome composition in severe COVID-19 cases was mentioned in two studies. In severe cases, fourteen Microviridae phages, one Inoviridae phage, one Podoviridae phage, and one unclassified virus were increased and plant-derived RNA virus, pepper chlorotic spot virus (PCSV), Myxococcus phage, Rheinheimera phage, Microcystis virus, Bacteroides phage, Murmansk poxvirus, Saudi moumouvirus, Sphaerotilus phage, Tomelloso virus, and Ruegeria phage were decreased significantly. See **Table 4**.

One study evaluated the associations between gut microbiota disturbance and SARS-CoV-2 viral loads and revealed that *Prevotella copri* and *Eubacterium dolichum* were positively correlated and *Streptococcus anginosus*, *Dialister* spp., *Alistipes* spp., *Ruminococcus* spp., *Clostridium citroniae*, *Bifidobacterium* spp., *Haemophilus* spp., and *Haemophilus parainfluenzae* were negatively correlated with the viral load of SARS-CoV-2.

### Biochemical and Immunologic Modifications in Relation to Gut Microbiota Alternations in COVID-19 Patients

In most studies, compared with healthy controls, COVID-19 patients had significantly higher levels of interleukin (IL)-2, IL-4, IL-6, IL-10, tumor necrosis factor **(**TNF)-α, and C-reactive protein (CRP) and lower lymphocyte counts.

According to one study, a positive correlation between *Bifidobacterium* spp. and prothrombin time (PT) and lactate dehydrogenase (LDH) was shown. Also, a negative correlation was reported between *Atopobium* spp. and D-dimer, *Bacteroides* spp. and LDH and creatine kinase (CK) level, *Clostridium butyricum*, and CRP and neutrophil count, and *Faecalibacterium prausnitzii* and CRP in critical COVID-19 patients. One study showed a specific relation between some genus of gut microbiota and immunological and biochemical modifications in critical and severe COVID-19 patients. In severe patients, *Faecalibacterium prausnitzii* and *Clostridium leptum* had a positive correlation with neutrophil count as well as *Eubacterium rectale* with IL-6 and Enterobacteriaceae with AST.

Another study indicated the specific relation of some species of gut microbiome and immune cells as the following: *Bacteroides ovatus*, *Lachnospiraceae* bacterium, and *Eubacterium ventriosum* had a positive correlation with CD4 and CD8 lymphocytes and other T-cells, in contrast to *Bifidobacterium animalis* and *Escherichia* spp. On the other hand, *Faecalibacterium prausnitzii* had a positive correlation with NK cells and *Coprobacillus* spp., *Clostridium ramosum*, and *Clostridium symbiosum* had a negative correlation with them.

### Studied Value of Gut Microbiome in COVID-19

All of the included studies showed a correlation between intestinal microbiota and COVID-19, and they studied the correlation in different aspects as in the following.

Four studies suggested that microbiota could have therapeutic properties with reducing gastrointestinal (GI) symptoms. *Streptococcus*, *Lactobacillus*, and *Bifidobacterium* were the most common bacterial genera interventions used so far. Nine articles demonstrated intestinal microbiota modifications in infected cases with COVID-19 in which two of them confirmed the value of specified gut microbiota as a diagnostic tool and one of them studied gut microbiota changes during recovery time. Lachnospiraceae are a large family including *Fusicathenibacter*, *Eubacterium hallii* group, and *Roseburia*, and the Ruminococcaceae family including *Faecalibacterium prausnitzii* and *Ruminococcus* as well as *Clostridium* spp., *Bacteroides* spp., *Lactobacillus* spp., *Rothia* spp., *Actinomyces* spp., *Lactobacillus* spp., and *Streptococcus* spp. were the most common bacteria with diagnostic value. Thirteen studies demonstrated a relationship between gut microbiota changes and the intensity and prognosis of COVID-19. *Eubacterium*, *Faecalibacterium*, *Ruminococcus*, *Bacteroides*, *Clostridium, Lactobacillus, Bifidobacterium*, and *Roseburia* were the most notable bacterial genera with prognostic values. Details are shown in **Table 3**.

## Discussion

The interaction between gut microbiota and viral respiratory diseases such as COVID-19 is a complex, bilateral, and dynamic association. The current study emphasizes the role of the gut–lung axis (GLA) in the pathogenesis of COVID-19. One of the important aspects of GLA is the impact of gut microbiota on the supply and maintenance of the lung immune system, and its correlation with respiratory diseases and infections (He et al., 2017; Dang and Marsland, 2019; Ahmadi Badi et al., 2021; Allali et al., 2021). The gut and lung microbiome are closely related in health or disease conditions (Dickson and Huffnagle, 2015; Enaud et al., 2020; Ahmadi Badi et al., 2021). SARS-CoV-2 may cause a dysbiosis in the lung microbiota to increase the population of inflammatory bacteria such as *Klebsiella oxytoca* and *Rothia mucilaginosa* which is associated with acute respiratory distress syndrome (Aktaş and Aslim, 2020; Han et al., 2020; van der Lelie and Taghavi, 2020; Battaglini et al., 2021; Chattopadhyay and Shankar, 2021). The high levels of inflammatory cytokine productions during SARS-CoV-2 invasion interfere with gut mucosal integrity and increasing risk of bacterial disposition to the bloodstream (Chattopadhyay and Shankar, 2021; Prasad et al., 2021).

Based on our findings, gut microbiota in patients with SARS-CoV-2 is significantly affected, possibly due to systemic inflammatory response. Although the underlying mechanism for the observed dysbiosis is unclear, it might happen *via* downregulation of ACE2 expression that alleviates the intestinal absorption of tryptophan leading to decreased secretion of antimicrobial peptides and changes the composition of gut microbiota (He et al., 2020). In a healthy individual, intact bacteria and their fragments or metabolites such as des-amino-tyrosine and short-chain fatty acids (SCFAs) pass across the intestinal barrier via the mesenteric lymphatic system, reach the lung, and activate the innate immune system by the production of cytokines like type 1 interferon (IFN1) (Antunes et al., 2019). In the current study, *Faecalibacterium prausnitzii* and *Clostridium leptum* have a positive correlation with neutrophil counts in COVID-19 patients and a negative correlation with *Clostridium butyricum*. In addition to the innate immunity, gut microbiota improves the function of CD8+ T-cell effectors (Trompette et al., 2018). There is also evidence that few bacterial species such as *Bacteroides ovatus*, *Lachnospiraceae* bacterium 5_1_63FAA, and *Eubacterium ventriosum* have an anti-inflammatory property in CD4^+^ and CD8^+^ T cells (Cao et al., 2021). Natural killer (NK) cells and B cells are also affected by gut microbiota; *Coprobacillus* spp., *Clostridium ramosum*, and *Clostridium symbiosum* are negatively associated with NK cell activity and *Bacteroides uniformis*, *Faecalibacterium prausnitzii*, and *Subdoligranulum* are positively correlated with B cells (Cao et al., 2021).

We found that the dysbiosis of gut microbiota may be a determinant factor in the clinical severity of COVID-19. Increase of the dominant *Enterococcus* and reduction of Ruminococcaceae and Lachnospiraceae are reported in severe cases of COVID-19 who were admitted to the medical intensive unit (MICU) (Gaibani et al., 2021).

Diversity of gut microbiota reduces in patients with COVID-19, which is associated with pro-inflammatory reaction and increased risk of opportunistic infections. GI symptoms of COVID-19 are reported to be strongly affected by gut microbial composition (Din et al., 2021; Katz-Agranov and Zandman-Goddard, 2021). The expression of the ACE-2 receptor on the surface of small intestine epithelial cells has been significantly associated with the extent of GI symptoms and fecal viral shedding during the course of disease (D’Amico et al., 2020; Patel et al., 2020; Wu et al., 2020; Katz-Agranov and Zandman-Goddard, 2021; Suárez-Fariñas et al., 2021). An increased expression of ACE-2 receptor in COVID-19 may occur due to a dominance of *Coprobacillus* in gut microbiota (Geva-Zatorsky et al., 2017; Enaud et al., 2020; Zuo et al., 2020a; Massip Copiz, 2021; Rajput et al., 2021; Walton et al., 2021). It is also noteworthy to imply that the expression of ACE-2 on luminal cells may be a determinant factor in microbial composition as the ACE-2 receptor plays some role in amino-acid absorption (Harmer et al., 2002; Dang and Marsland, 2019).

*Eggerthella* is another genus of bacteria which significantly increases in patients with COVID-19 (d’Ettorre et al., 2020; Cao et al., 2021). *Eggerthella* may induce colitis *via* abnormal activation of Th17 in patients with inflammatory diseases. It can interfere with gut integrity and make the patient more susceptible to pathogen invasion including SARS-CoV-2 (Alexander et al., 2019; Katz-Agranov and Zandman-Goddard, 2021).

There is a dominancy of genus *Clostridium* in patients with COVID-19 (Gu et al., 2020b; Zuo et al., 2020a; Cao et al., 2021; Yamamoto et al., 2021). The increase in *Clostridium ramosum* and *Clostridium hathewayi* is associated with the disease severity which may be a risk factor of acute portal vein thrombosis (Zuo et al., 2020; Rokkam et al., 2021). *Clostridium difficile* may complicate COVID-19 and worsen the GI symptoms (Ferreira et al., 2020; Oba et al., 2020; Sandhu et al., 2020; Khanna and Kraft, 2021). Two butyrate-producing members of this genus, *Clostridium butyricum* and *Clostridium leptum*, are decreased in patients with COVID-19 (Tang et al., 2020).

*Streptococcus* is another important bacterial genus which increases in COVID-19 (d’Ettorre et al., 2020; Donati Zeppa et al., 2020; Gu et al., 2020b; Zuo et al., 2021a). The abundance of *Streptococcus* is also an indicator of the extent of opportunistic bacterial invasion (Weiser et al., 2018; Tao et al., 2020). *Streptococcus* abundance is associated with more expressions of IL-18, TNF-α, and IFN-γ and other inflammatory cytokines worsening clinical outcomes (Tao et al., 2020; van der Lelie and Taghavi, 2020; Chhibber-Goel et al., 2021). An altered gut integrity affected by dysbiosis and inflammatory cytokines seems to be the main cause of increase in *Streptococcus* abundance in COVID-19 (Donati Zeppa et al., 2020). *Streptococcus* also affects the lung microbiome with proinflammatory activity (Kyo et al., 2019; Yamamoto et al., 2021).

Genus *Rothia* dominancy increases in patients with COVID-19 (Gu et al., 2020b; Lv et al., 2021a). This genus seems to be associated with inflammatory lung injuries (Han et al., 2020; Chattopadhyay and Shankar, 2021).

The genus *Collinsella* is an opportunistic pathogenic genus which is widely found in the gut of patients with COVID-19 especially in severe cases and higher infectivity status (Chhibber-Goel et al., 2021; Liu et al., 2021; Massip Copiz, 2021; Rajput et al., 2021; Zuo et al., 2021a). *Collinsella aerofaciens* abundance altered with the gut mucosal integrity and production of inflammatory mediators such as IL-17, CXCL1, and CXCL5 from the luminal cells (Kalinkovich and Livshits, 2019).

The alteration of genus *Parabacteroides* in COVID-19 is an area of controversy (Tang et al., 2020; Tao et al., 2020; Chhibber-Goel et al., 2021; Zuo et al., 2021a; Yeoh et al., 2021). It has been suggested that higher levels of *Parabacteroides* in microbiota are associated with better gut mucosal integrity (Venegas et al., 2019; Tang et al., 2020; Chhibber-Goel et al., 2021).

*Ruminococcus* species such as *Ruminococcus gnavus* and *Ruminococcus torques* increase (Cao et al., 2021; Yeoh et al., 2021), and species including *Ruminococcus bromii*, *Ruminococcus obeum*, and *Ruminococcus* sp. 5139BFAA are reduced in patients with COVID-19 (Zuo et al., 2020a; Cao et al., 2021; Lv et al., 2021). *Ruminococcus gnavus* and *Ruminococcus torques* are known as proinflammatory bacteria which previously have been shown to be associated with proinflammatory status and higher production of inflammatory mediators (Matsuoka and Kanai, 2015; Hall et al., 2017; Henke et al., 2019; Yeoh et al., 2021). Reduction of *Ruminococcus obeum* seems to be secondary to the wide use/misuse of antibiotics in the management of COVID-19 patients (Cyprian et al., 2021). Among *Alistipes* genera, *Alistipes*_*sp*_*AP11*, *Alistipes indistinctus*, and *Alistipes shahii* are reduced and *Alistipes onderdonkii* increased in COVID-19 (Zuo et al., 2020a; Cao et al., 2021). *Alistipes onderdonkii* is one of the most important sources of short-chain fatty acid (SCFA) production in the gut that helps to the gut homeostasis (Venegas et al., 2019; Tang et al., 2020). *Alistipes* is also important in preservation of the gut immunity *via* being involved in tryptophan synthesis pathways (Gao et al., 2018).

*Bacteroides alteration* is reported in COVID-19, especially in critically ill patients (Tang et al., 2020; Cao et al., 2021; Chattopadhyay and Shankar, 2021; Yeoh et al., 2021; Zuo et al., 2021a). *Bacteroides* are the most critical commensal bacterial genera in gut whose alterations are associated with several conditions affecting human health and disease (Ley et al., 2006; Turnbaugh et al., 2006; Larsen et al., 2010; Claesson et al., 2012; Yu et al., 2015; Boursier et al., 2016; Salazar et al., 2017; Belizário et al., 2018; O’Toole and Jeffery, 2018; Yildiz et al., 2018; Antosca et al., 2019; Rahayu et al., 2019; Crovesy et al., 2020; Juárez-Fernández et al., 2021). *Bacteroides* also have immunomodulatory effects, which is mainly mediated by alterations in production of polysaccharide A, IL-6, IL-7, IL-10, dendritic cells, and CD4+ and CD8+ T cells (Abt et al., 2012; Zhang et al., 2018; Jia et al., 2018; Ramakrishna et al., 2019; Alvarez et al., 2020; Gautier et al., 2021). *Bacteroides* are also associated with reduction of the expression of the ACE-2 receptor (Chattopadhyay and Shankar, 2021). Use of antibiotics seems to result in increase of *Bacteroides caccae* in a COVID-19 patient. In antibiotic-naive COVID-19 patients, *Bacteroides nordii* are more common (Chattopadhyay and Shankar, 2021). On the other hand, species such as *Bacteroides massiliensis*, *Bacteroides dorei*, *Bacteroides thetaiotaomicron*, and *Bacteroides ovatus* decrease in SARS-CoV-2 infection (Zuo et al., 2020a; Cao et al., 2021). *Bacteroides dorei* itself is a controversial bacterium with both evidence of increase and decrease in COVID-19 patients. This species is associated with IL-6 and IL-8 and downregulation of the ACE-2 receptor (Yoshida et al., 2018; Yeoh et al., 2021).

*Bifidobacterium*, a major bacterial genus in the gut, reduces by SARS-CoV-2 (Gu et al., 2020b). In patients who received fecal microbial transplant (FMT), a re-expansion of *Bifidobacterium* in their gut was demonstrated (Liu et al., 2021). *Bifidobacterium* spp. are well known for their immunomodulatory effects especially on Th17 and in the amelioration of inflammatory process (de Vrese et al., 2005; Wang et al., 2011; Groeger et al., 2013; Jungersen et al., 2014; King et al., 2014; Han et al., 2016; Schiavi et al., 2016; Bozkurt et al., 2019; Bozkurt and Kara, 2020; Tian et al., 2020; Arenas-Padilla et al., 2021; Chen and Vitetta, 2021; Hong et al., 2021; Milner et al., 2021). *Bifidobacterium* may be considered as a supplemental therapeutic agent for controlling cytokine storm and inflammation in patients with COVID-19 (Bozkurt and Quigley, 2020; Bozkurt and Quigley, 2020a; Schett et al., 2020; Bhushan et al., 2021 Gautier et al., 2021; Mohseni et al., 2021).

*Faecalibacterium* decreases in COVID-19 and has been related to the severity of disease (Zuo et al., 2020; Gu et al., 2020; Tang et al., 2020; Yamamoto et al., 2021; Lv et al., 2021). *Faecalibacterium* is a butyrate-producing genus which positively impacts on intestinal mucosal integrity and is also known to have anti-inflammatory effects (Alameddine et al., 2019; Zuo et al., 2020a; Chhibber-Goel et al., 2021). Fecal transplantation significantly increases the abundance of *Faecalibacterium* in discharged patients with COVID-19 and has been shown to improve the inflammation states (Sokol et al., 2008; van den Munckhof et al., 2018; Venegas et al., 2019; Liu et al., 2021).

Lachnospiraceae, which is known as SCFA-producing bacteria, decreases in patients with COVID-19 (d’Ettorre et al., 2020; Gu et al., 2020b; Zuo et al., 2020a; Cao et al., 2021; Gaibani et al., 2021; Gautier et al., 2021; Zuo et al., 2021a). It may be attributed to common use of azithromycin and other antibiotics in the management of COVID-19 (Segal et al., 2020).

Genus *Roseburia* is another commensal gut microbiota which decreases in patients with COVID-19 and other viral diseases such as influenza (Wang et al., 2017; Gu et al., 2020b; Cao et al., 2021; Lv et al., 2021a). SCFAs produced by *Roseburia* maintain mucosal integrity in healthy adults *via* modulation of inflammatory mediators especially IL-10 (Koh et al., 2016; Zheng et al., 2017; Haak et al., 2018; Gautier et al., 2021). It has been shown previously that butyrate may preserve lung integrity from cytokine-induced injuries in influenza (Chakraborty et al., 2017; Dang and Marsland, 2019). If we assume that it is true in COVID-19, lower levels of *Roseburia* result in lower levels of butyrate and consequently more extensive lung injuries due to inflammatory processes.

*Eubacterium* is a genus with immunomodulatory effects which significantly decrease in gut microbiota of patients with COVID-19 (d’Ettorre et al., 2020; Zuo et al., 2020a; Gu et al., 2020b; Cao et al., 2021; Chattopadhyay and Shankar, 2021; Gautier et al., 2021; Lv et al., 2021; Yeoh et al., 2021). Wide use of antibiotics is considered to be associated with reduction of this genus (Zuo et al., 2020a). This genus similar to *Roseburia* spp. produces butyrate and modulates inflammation in inflammation-mediated injuries (Koh et al., 2016; Zheng et al., 2017; Haak et al., 2018; Gautier et al., 2021).

*Fusicatenibacter* is another bacterial genus that reduced during the course of COVID-19 (Gu et al., 2020; Chattopadhyay and Shankar, 2021; Lv et al., 2021a). *Fusicatenibacter* alteration is a very sensitive biomarker during COVID-19. It is proposed to be a diagnostic tool for COVID-19 (Gu et al., 2020b; Segal et al., 2020; Cyprian et al., 2021; Howell et al., 2021). This genus is also negatively correlated with CRP and procalcitonin levels in patients with COVID-19 (Gu et al., 2020b).

Other members of the gut microbiota are viruses and fungi. Although pathogenic gut viruses are known for more than a century, the term “gut virome” is recently introduced (Reyes et al., 2012). Most of the gut virome consists of bacteriophages that can explain the fact that the virome structure is related to gut bacterial composition in both healthy and COVID-19 people (Minot et al., 2011; Cao et al., 2021). There is a bidirectional relationship between gut virome and infectious diseases; bacteriophages have a significant role in protecting against bacterial infections (Wilks and Golovkina, 2012). Gut virome composition might be affected during COVID-19 (Cao et al., 2021). There are limited data on the alterations of commensal viral and fungal populations in the gut during COVID-19 infection.

Lv et al. showed a strong correlation between altered fungal gut microbiome and inflammatory blood biomarkers (Lv et al., 2021b). Further studies focusing on viral and fungal alterations during the COVID-19 are desired.

The gut microbiota alteration in COVID-19 patients should be considered as a dynamic process (d’Ettorre et al., 2020; Liu et al., 2021; Hussain et al., 2021; Zuo et al., 2020a). To the date of revising this manuscript (January 2022), several registered clinical trials are in progress and the results are not provided yet; however, growing evidence supports the effectiveness of microbiota modulatory actions on fastening the recovery of patients with COVID-19 (Chen and Vitetta, 2021; Hussain et al., 2021; Wang et al., 2021a).

### Limitations and Suggestions

A few studies have documented the comorbidities of subjects. However, almost all the studies have missed the impact of comorbidities on gut microbiota alterations in COVID-19 patients compared with the healthy control. It has been shown that gut microbiota may change in patients with hypertension, cardiovascular diseases, diabetes mellitus, hyperlipidemia, and thrombotic events (Huynh, 2020; Avery et al., 2021; Kyriakidou et al., 2021; Mineshita et al., 2021). We strongly propose to investigate the effects of underlying comorbidities in gut microbial composition in patients with COVID-19.

Due to the variations in data analysis techniques such as 16S rRNA sequencing, qPCR, and metagenome sequencing, there was a challenge to compare the bacterial taxa across studies. Since there was a fair diversity in geographical distribution of the current studies (most of them are from China), we cannot ignore the effect of diet and genetic predisposing factors like HLA in gut microbiome compositions. Future studies in different countries are required in this regard.

It is important to mention that different levels of p-value significance were reported in reviewed articles; however, in this study we used only statistically significant findings from the included studies.

Further studies with a larger study population, including the range of patients from mild to severe symptoms, involving the patients who are managed out patiently, focusing on the effectiveness of gut microbiota-targeted therapies for prevention and improvement of COVID-19 patients’ symptoms are desired to light up this topic.

## Conclusion

Our study showed a significant alteration of gut microbiome composition in patients with COVID-19 compared to healthy individuals. This great extent of impact has proposed the gut microbiota as a potential diagnostic, prognostic, and potentially therapeutic strategy for COVID-19.

## Author Contributions

MN and PJ designed the study. MN, YF, AT, PJ, MA, and FV performed the search and data extraction and wrote the first draft of the manuscript. LS, AHSB, and MM reviewed and revised the manuscript. All authors contributed to the article and approved the submitted version.

## Conflict of Interest

The authors declare that the research was conducted in the absence of any commercial or financial relationships that could be construed as a potential conflict of interest.

## Publisher’s Note

All claims expressed in this article are solely those of the authors and do not necessarily represent those of their affiliated organizations, or those of the publisher, the editors and the reviewers. Any product that may be evaluated in this article, or claim that may be made by its manufacturer, is not guaranteed or endorsed by the publisher.
